# Protective effect of hydroxytyrosol and tyrosol metabolites in LPS-induced vascular barrier derangement *in vitro*

**DOI:** 10.3389/fnut.2024.1350378

**Published:** 2024-04-19

**Authors:** Sonia Zodio, Gabriele Serreli, Maria Paola Melis, Benedetta Franchi, Anna Boronat, Rafael de la Torre, Monica Deiana

**Affiliations:** ^1^Department of Biomedical Sciences, University of Cagliari, Cagliari, Italy; ^2^Department of Medicine and Life Sciences, Universitat Pompeu Fabra, Barcelona, Spain; ^3^Integrative Pharmacology and Systems Neurosciences Research Group, Hospital del Mar Research Institute, Barcelona, Spain; ^4^Physiopathology of Obesity and Nutrition Networking Biomedical Research Centre (CIBEROBN), Madrid, Spain

**Keywords:** endothelial dysfunction, extra virgin olive oil, inflammation, phenolic compounds, tight junctions

## Abstract

**Introduction:**

The maintenance of endothelial barrier function is essential for vasal homeostasis and prevention of cardiovascular diseases. Among the toxic stimuli involved in the initiation of atherosclerotic lesions, Gram negative lipopolysaccharide (LPS) has been reported to be able to trigger endothelial dysfunction, through the alteration of barrier permeability and inflammatory response. Hydroxytyrosol (HT) and tyrosol (Tyr), the major phenolic compounds of extra virgin olive oil (EVOO), as wells as their circulating sulphated and glucuronidated metabolites have been shown to exert anti-inflammatory effects at endothelial level.

**Methods:**

In this study we investigated the protective effects of HT and Tyr metabolites on LPS-induced alteration of permeability in Human Umbilical Vein Endothelial Cells (HUVEC) monolayers and examined underlying signaling pathways, focusing on tight junction (TJ) proteins, mitogen-activated protein kinase (MAPK) and NOD-, LRR-and pyrin domain-containing protein 3 (NLRP3) inflammasome activation.

**Results:**

It was shown that LPS-increased permeability in HUVEC cells was due to the alteration of TJ protein level, following the activation of MAPK and NLRP3. HT and Tyr sulphated and glucuronidated metabolites were able to limit the effects exerted by LPS, acting as signaling molecules with an efficacy comparable to that of their precursors HT and Tyr.

**Discussion:**

The obtained results add a further piece to the understanding of HT and Tyr metabolites mechanisms of action in vascular protection.

## 1 Introduction

Lipopolysaccharide (LPS) is an endotoxin found in the outer membrane of Gram-negative bacteria in large quantities in the human colon. Small amounts of LPS continually translocate from the gut lumen to the circulation, the so-called metabolic endotoxemia (1), and are correlated to the incidence or prevalence of atherosclerosis (2). A key event in the vascular dysfunction involved in the atherosclerotic process led by LPS is the breakdown of barrier function that results in a profound increase in permeability (3). This process involves adhesive cell–cell junctional proteins, like the tight junctions (TJ), that consist of the interaction of the proteins occludin and claudin with the intracellular zonula occludens (ZO) 1, 2, or 3 (4) and junctional adhesion molecule (JAM) (5). LPS has been proposed to induce the formation of paracellular gaps and cytoskeletal rearrangement (6) and to interact with TJ proteins (7) through the modulation of inflammatory signaling pathways (8–10). LPS-mediated vascular inflammatory responses could be modulated by dietary anti-inflammatory agents such as phenolic compounds (11). Hydroxytyrosol (HT) and tyrosol (Tyr), peculiar compounds of extra virgin olive oil (EVOO), have been widely studied for their potential beneficial effects in the prevention of cardiovascular diseases (12), linked to their antioxidant and anti-inflammatory abilities (13). HT and Tyr are well absorbed and undergo an extensive metabolization, through phase I and phase II reactions, originating mainly sulphated and glucuronidated forms (14). These metabolites reach higher circulating concentrations than those of unmetabolized Tyr and HT, thus possibly contributing their biological effect observed at vascular level (15). In this study we investigated the ability of HT and Tyr major metabolites, Tyr-glucuronide, Tyr sulfate, 3′- HT-glucuronide and HT-3-sulfate (Supplementary Figure S1), to modulate LPS-induced inflammatory response in HUVEC (Human Umbilical Vein Endothelial Cells), as a model of vascular endothelium. Metabolites, at physiologically dietary relevant concentrations, were tested to investigate their ability to maintain barrier integrity. For a deeper insight into the molecular mechanisms involved, we evaluated the modulation of TJ proteins occludin, ZO-1 and JAM-A and upstream cellular signaling pathways, as MAPK (mitogen-activated protein kinase) and NLRP3 (NOD-, LRR-and pyrin domain-containing protein 3) inflammasome.

## 2 Materials and methods

### 2.1 Chemicals and reagents

Bovine Serum Albumin, fluorescein isothiocyanate-dextran (wt 4,000), dimethyl-sulfoxide (DMSO), Bradford reagent, Cell Lytic-M, lipopolysaccharide from *Escherichia coli*, hydroxytyrosol, tyrosol, NaCl, NaF, K_2_HPO_4_, KH_2_PO_4_, MgSO_4_x7H_2_O, CaCl_2_x6H_2_O, NaHCO_3_, Tween 80 and all solvents of analytical grade were purchased from Sigma Aldrich (Milano, Italy). Tyrosol glucuronide, tyrosol sulfate sodium salt, 3′- hydroxytyrosol 3′-glucuronide, hydroxytyrosol 3-sulfate sodium salt, were obtained from LGC standards (Sesto San Giovanni, Italy). The Phosphatase and Protease Inhibitor Cocktail, nitrocellulose membranes, gels and all material for electrophoresis were purchased from ThermoFisher Scientific (Massachusetts, United States). ReagentPack Subculture Reagents with Trypsin/EDTA, TNS (Trypsin Neutralizer solution) and HEPES (2-[4-(2-hydroxyethyl)piperazin-1-yl]ethanesulfonic acid) solutions were obtained from Lonza (Basel, Switzerland). Transwell inserts were obtained from Corning Costar Corp. (New York, N.Y., United States).

### 2.2 Cell line cultures

HUVEC cells were obtained from Lonza (Basel, Switzerland) and were grown until confluence in EBM-2 supplemented with 2% FBS, 0.2% heparin, 0.2% hydrocortisone, 0.2% hFGFb (Human Fibroblast Growth Factor basic), 0.2% hVEGF (Human Vascular Endothelial Growth Factor), 0.2% long R3-IGF-1 (analog of Human Insulin-Like Growth Factor-1), 0.2% ascorbic acid, 0.2% hEGF (Human Epidermal Growth Factor) and 0.2% of GA 1000 (gentamycin sulfate) (BulletKit^™^) obtained from Lonza (Basel, Switzerland).

HUVEC cells were maintained at 37°C in a 5% CO_2_ humidified atmosphere. At passage 1–5, cells were removed from flasks and then seeded into 6 well, 96 well or transwell plates at a concentration of 5 × 10^4^/mL for different experiments, replacing the medium twice a week.

### 2.3 MTT assay

The MTT (3-(4,5-dimethylthiazol-2-yl)-2,5-diphenyltetrazolium bromide) assay was performed as reported by Serreli et al. (16). Briefly, cells were exposed to a range of concentrations of the compounds (0.5–2.5 μM, in serum free medium), or an equivalent volume of vehicle (MeOH) for the controls (0 μM), and incubated for 24 h with or without LPS (10 μg/mL). After the treatments, the medium was replaced by 100 μL of MTT solution (0.5 mg/mL in supplemented growth media) and left for 4 h at 37°C. The medium was removed, 100 μL of DMSO were added in each well and the absorbance was read at 570 nm by using a Multiskan Ex microplate reader (Thermo Fisher Scientific, Paisley, UK). After subtracting the blank values, data were converted to % of cells viability as follows: % cell viability = Abs sample/Abs control × 100.

### 2.4 FITC-dextran permeability assay

HUVECs (1 × 10^5^ cells/mL) were seeded on transwell filters (0.4-μm pore size, Costar, New York, USA) in 12-well dishes and grown until confluence. In the first experimental set, cells were incubated with LPS (10 μg/mL) for 2 h, 4 h, 6 h and 18 h to evaluate the effects induced by LPS over time. In the second set, cells were pretreated with HT, Tyr, and their metabolites (1 μM) or with an equivalent volume of vehicle (MeOH) for the controls (0 μM) for 30 min prior to incubation with LPS for 2 h. After treatment, the medium was replaced with Fluorescein isothiocyanate (FITC)-dextran solution in the upper chamber at a final concentration of 1 mg/mL in medium. After 1 h of incubation at 37° C, paracellular flux was assessed by taking 100 μL aliquots from the lower chamber to measure real-time changes of permeability across endothelial cell monolayers (17). Fluorescence was measured in collected samples using a fluorescence plate reader at excitation and emission wavelengths of 485 and 530 nm, respectively. The concentration of basal permeable FITC-Dextran was calculated compared to control samples and each sample was performed in triplicate.

### 2.5 Western blot analyses

HUVEC cells were seeded in 6-well plates (5×10^4^ cells/mL in 2 mL of growth media) for one week and then treated with LPS (10 μg/mL) alone for different incubation times (1 h, 2 h, 3 h, 4 h, 5 h, 6 h, 18 h and 24 h), or together with Tyr, HT and their sulphated and glucuronidated metabolites (1 μM) for 2 h before adding the LPS solution (10 μg/mL) for 3 h for MAPK and TJ proteins determination. An equivalent volume of vehicle (MeOH) was added to control cells. The medium was then removed and 180 μL lysis buffer (supplemented with phosphatase and protease inhibitor) was added. The cell lysate was placed in Eppendorf tubes, centrifuged at 12500 rpm for 7 min and the supernatant recovered. Protein concentration was determined following the Bradford protocol (18). Denatured proteins (20–50 μg depending on the protein) were separated using 4–12, 10% and 4–20% polyacrylamide gel, then transferred into nitrocellulose membrane where they were blocked with 25 mL of a TBS (Tris/HCl, pH 7.5, 100 mM NaCl) and 4% milk solution for 40 min. After washing with TBS solution, membranes where incubated overnight, at 4°C, with primary polyclonal antibodies, anti-ERK1/2 (ab184699), anti-phospho ERK1/2 (ab278538), anti-p38 (ab170099), anti-phospho p38 (ab178867), anti-β actin (ab8226), anti-JAM-A (ab52647), anti-ZO-1 (ab276131), anti-occludin (ab216327) (Abcam, Cambridge, UK) and then washed twice with TTBS (TBS with Tween 20 0.5%) before adding the secondary anti-rabbit (AP132P) and/or anti-mouse (AP124P) antibody IgG peroxidase-conjugated (Sigma Aldrich, Milan, Italy). Both primary and secondary antibodies were prepare adding an aliquot of the original solution in 10 mL of TTBS solution with 1% of milk (dilution 1:1000 v/v). Membranes were washed twice with TTBS and one time with TBS, exposed to Clarity^™^Western-ECL (Bio-Rad) reagents (4–5 min) and observed through ChemiDoc^™^MT System. Analysis of the images obtained from ChemiDoc were analyzed using Quantity One (Biorad, Hemel Hempstead UK) software in order to determine the molecular weight of the protein bands, through the comparison with bands obtained by separation of a marker run together with proteins.

### 2.6 Statistical analysis

The statistical analysis was performed using the mean ± standard deviations for each of the groups in all the experiments (each experiment was performed at least 3 times); statistical significance within sets of data was determined by the analysis of variance “one-way ANOVA” and post-hoc Tukey’s test, with the software GraphPad Prism 5 (GraphPad software, San Diego, CA, United States).

## 3 Results

### 3.1 Cell viability

Cell viability was assessed in HUVEC cultures to evaluate the possible cytotoxic effects of the tested compounds (0.5–2.5 μM) incubated alone or with LPS (10 μg/mL) co-incubation. The reduction of the tetrazolium dye MTT, 3-(4,5-dimethylthiazol-2-yl)-2,5-diphenyltetrazolium bromide, to the insoluble formazan reflected the number of viable cells present. The cell viability was measured spectrophotometrically and expressed as a percentage of viability compared to the control (100% viability). The tested compounds did not affect significantly HUVEC cell viability at any concentration (*p* > 0.05) after 24 h of incubation. The same went for the compounds tested together with LPS, whose incubation did not result in any significant change in cell viability (Supplementary Figures S2, S3).

### 3.2 FITC-dextran permeability assay

**Figure 1:**
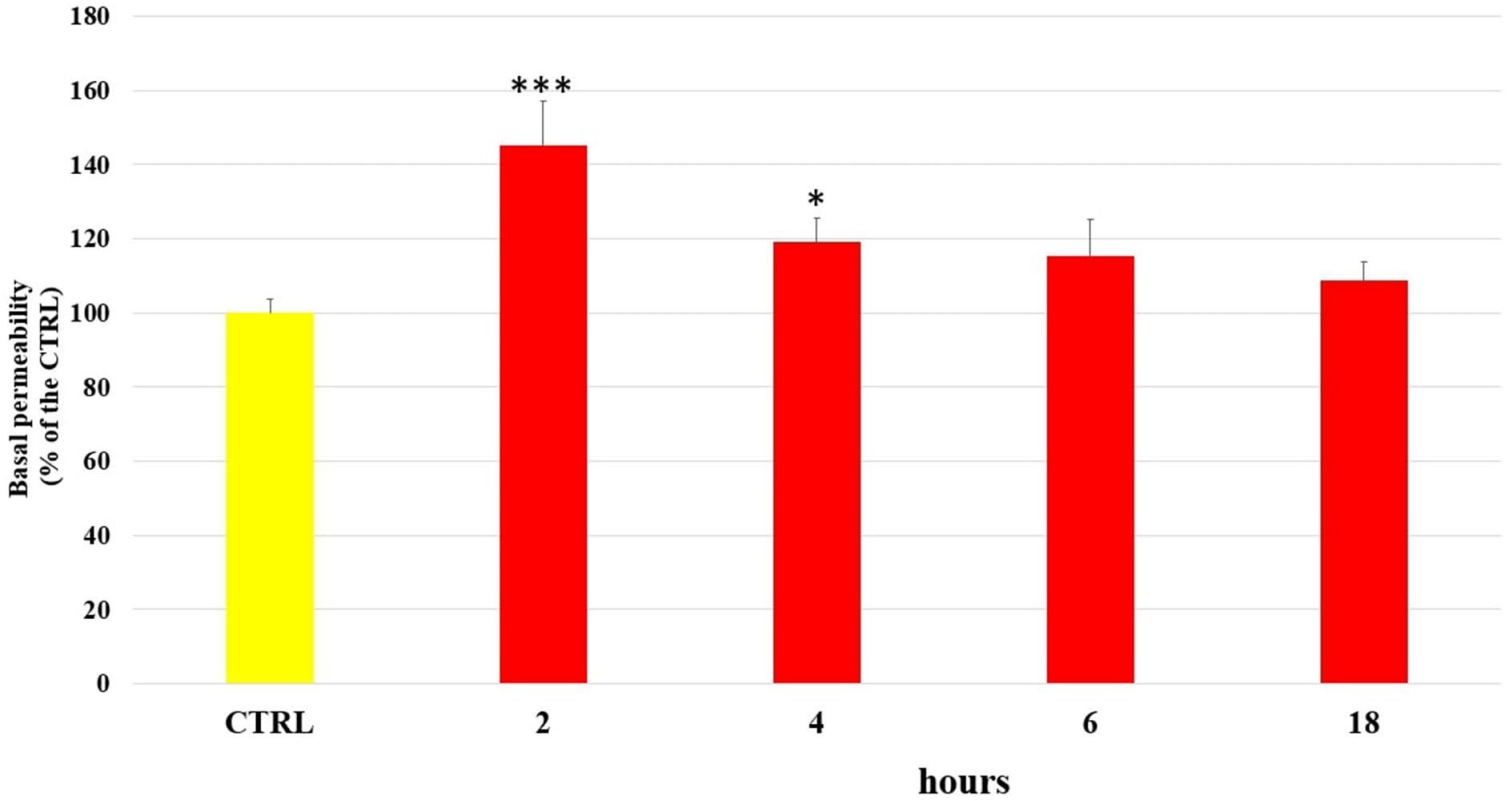
FITC-Dextran basal permeability measured in untreated HUVEC cells (CTRL) or treated with LPS (10 μg/mL) at 2 h, 4 h, 6 h and 18 h incubation times. Data are reported as percentage compared to CTRL for each time. * = *p* < 0.05; *** = *p* < 0.001 LPS vs. CTRL; (*n* = 3).

**Figure 2:**
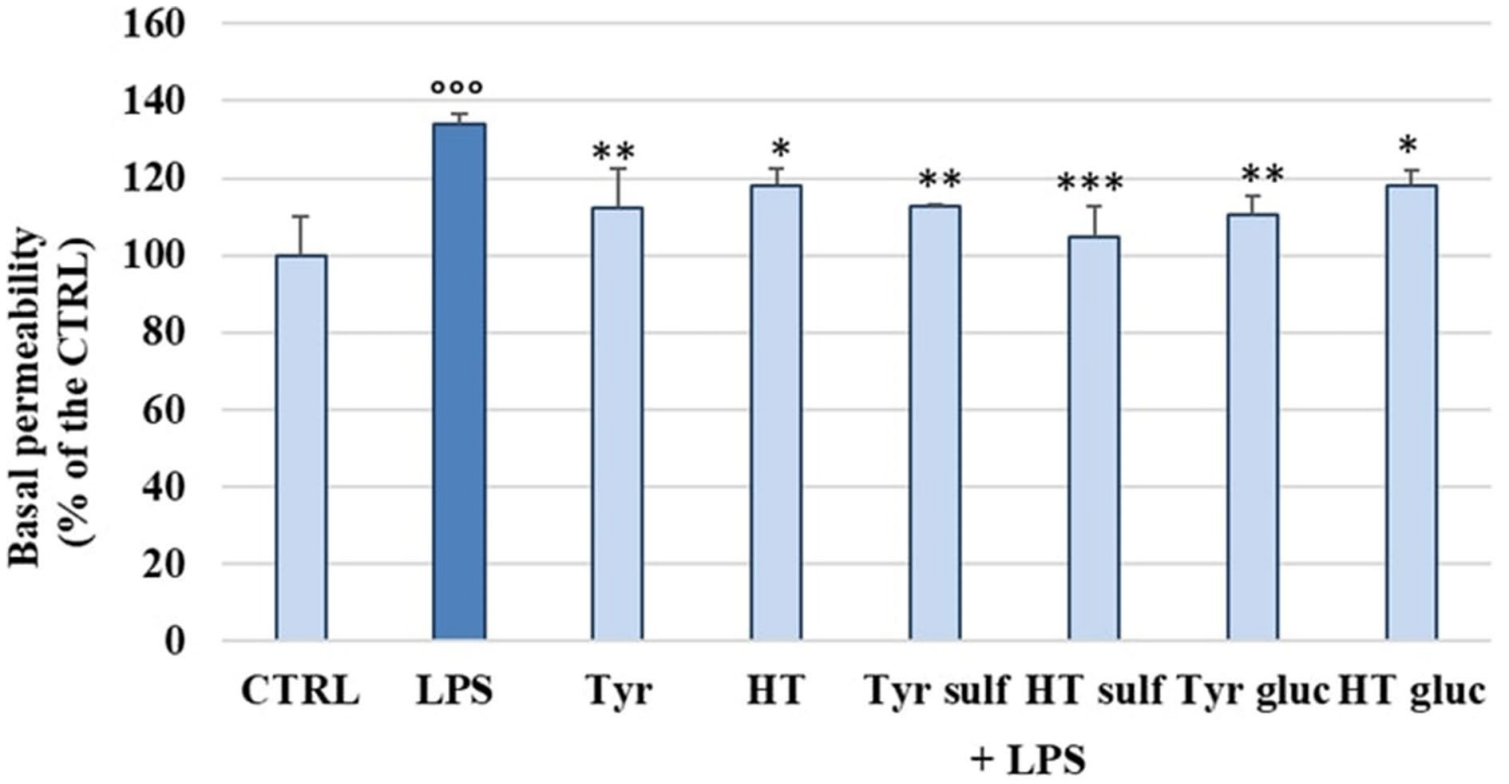
FITC-Dextran basal permeability measured in HUVEC cells pretreated with Tyr, HT, Tyr sulf, HT sulf, Tyr gluc, HT gluc (1 μM) or with an equivalent volume of MeOH (CTRL, 0 μM), and treated with LPS (10 μg/mL) for 2 h. Data are reported as percentage compared to CTRL for each sample. °°° = *p* < 0.001 LPS vs. CTRL; * = *p* < 0.05; ** = *p* < 0.01; *** = *p* < 0.001 compounds vs. LPS (*n* = 3).

The damage caused by LPS on the endothelial barrier was evaluated *in vitro* in HUVEC monolayers, as alteration of permeability, by the fluorescein isothiocyanate–dextran flux (FITC-Dextran) assay. FITC-dextran is usually transported via the paracellular route through TJs. The cells were treated with LPS (10 μg/mL) and incubated for 2 h, 4 h, 6 h and 18 h, at the end of which the basal permeability of FITC-Dextran was measured spectrophotometrically. Then, in the same way, the ability of the phenolic compounds (1 μM) to limit the alteration of endothelial permeability induced by LPS was tested after 2 h of incubation. Figure 1 shows the changes in endothelial cell permeability induced by LPS treatment from 2 h until 18 h. LPS significantly increased basal permeability at 2 h of incubation, about 40–45% respect to the untreated cells (CTRL), highlighting the role of LPS in the alteration of endothelial TJ. However, after 4 h of incubation the basal permeability of FITC-Dextran started to decrease and at 18 h of incubation CTRL values were restored. In Figure 2 were reported the values that indicate how pretreatment with all tested phenolic compounds significantly limited the basal FITC-Dextran permeability rise compared to LPS alone after 2 h of incubation, where permeability was about 30% higher. This protective effect was observed almost equally for all tested compounds (*p* > 0.05).

### 3.3 Determination of occludin, ZO-1 and JAM-A level

**Figure 3:**
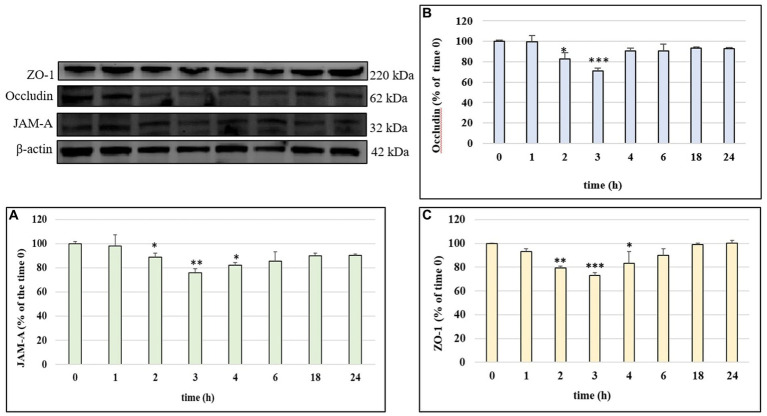
JAM-A **(A)**, occludin **(B)** and ZO-1 **(C)** /β-actin ratio measured in HUVEC cells before treatment (time 0) or treated with LPS 10 μg/mL at different incubation time. Data are reported as percentage compared to CTRL for each time. * = *p* < 0.05; ** = *p* < 0.01; *** = *p* < 0.001 LPS vs. CTRL; (*n* = 3). Representative WB images of the experiment are shown.

**Figure 4:**
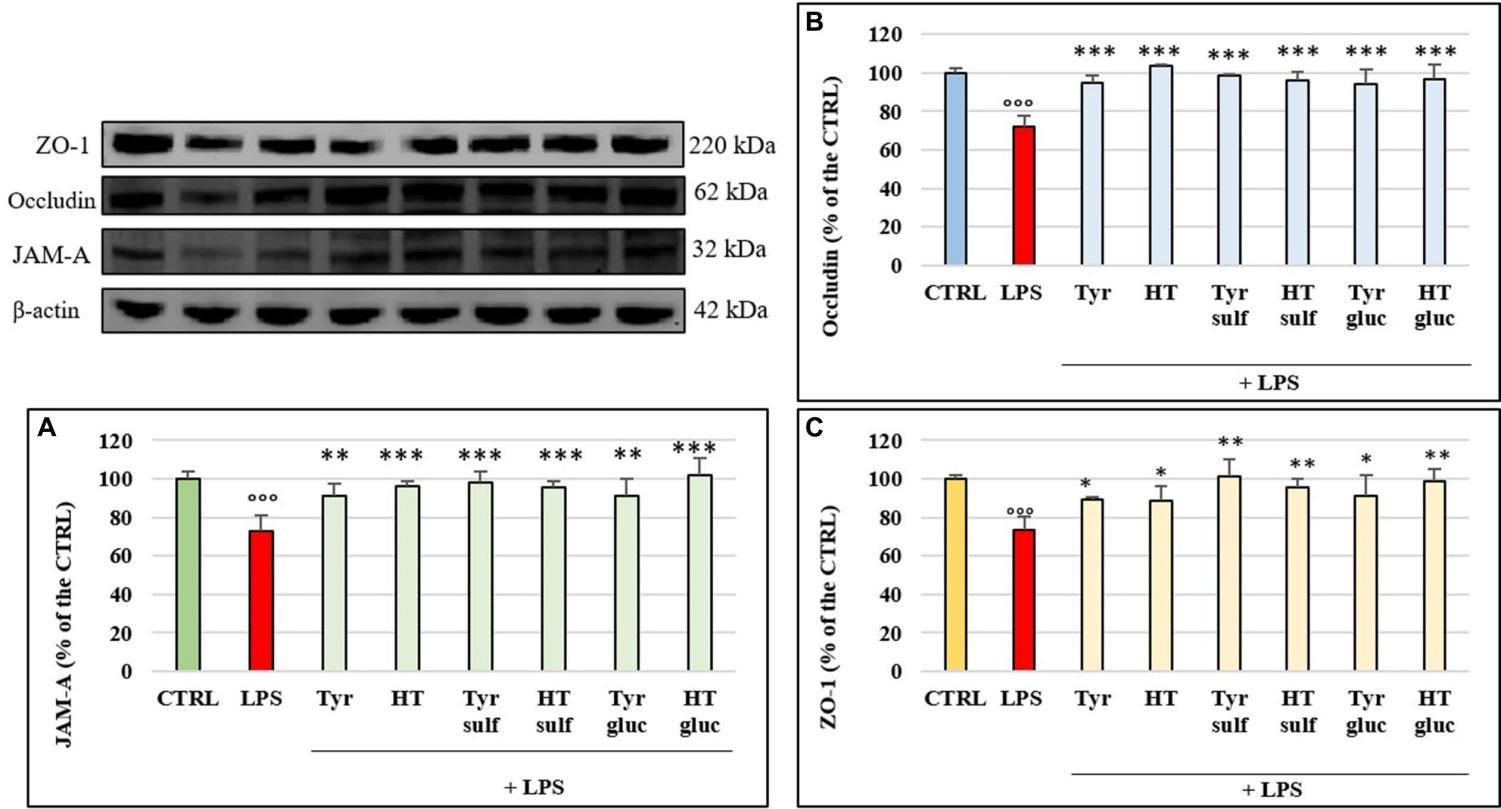
JAM-A **(A)**, occludin **(B)** and ZO-1 **(C)** /β-actin ratio measured in cells pretreated with Tyr, HT, Tyr sulf, HT sulf, Tyr gluc, HT gluc (1 μM) and treated with LPS (10 μg/mL) for 3 h of incubation. Data are reported as percentage compared to CTRL for each sample. °°° = *p* < 0.001 LPS vs. CTRL; * = *p* < 0.05; ** = *p* < 0.01; *** = *p* < 0.001 Compounds vs. LPS (*n* = 3). Representative WB images of the experiment are shown.

In order to investigate the mechanism of action by which LPS determined a significant alteration of permeability in HUVEC cell monolayers, we examined the modulation of TJ proteins occludin, ZO-1 and JAM-A with time, through Western blot analyses. The cells were treated with LPS (10 μg/mL) for 1, 2, 3, 4, 6, 18 and 24 h. Figures 3A–C shows the action of LPS on TJs integrity in HUVEC monolayers. The treatment with LPS led to a decrease in all three TJ proteins level compared to CTRL (100%). The highest decrease was observed at 3 h of incubation, with values about 25–30% less with respect to the CTRL. At 24 h, the concentration of all analyzed TJ proteins was restored to the CTRL values. The protective effect of phenolic compounds in LPS-induced TJ disruption was then evaluated. HUVEC were pre-treated with Tyr, Tyr sulf, Tyr gluc, HT, HT sulf and HT gluc and then incubated with LPS (10 μg/mL). Obtained data are reported in Figures 4A–C. After 3 h of incubation LPS was able to cause a decrease in TJ level of about 25–30% compared to CTRL (100%) (*p* < 0.001), while pretreatment with Tyr, HT and their sulphated and glucuronidated metabolites significantly preserved TJ proteins levels. Results were similar for all the evaluated TJ proteins, but, overall, the most significant results were observed for occludin (Figure 4B), where all compounds completely inhibited the decrease of protein concentration.

### 3.4 Modulation of MAPK p38 and ERK 1/2 activation

**Figure 5:**
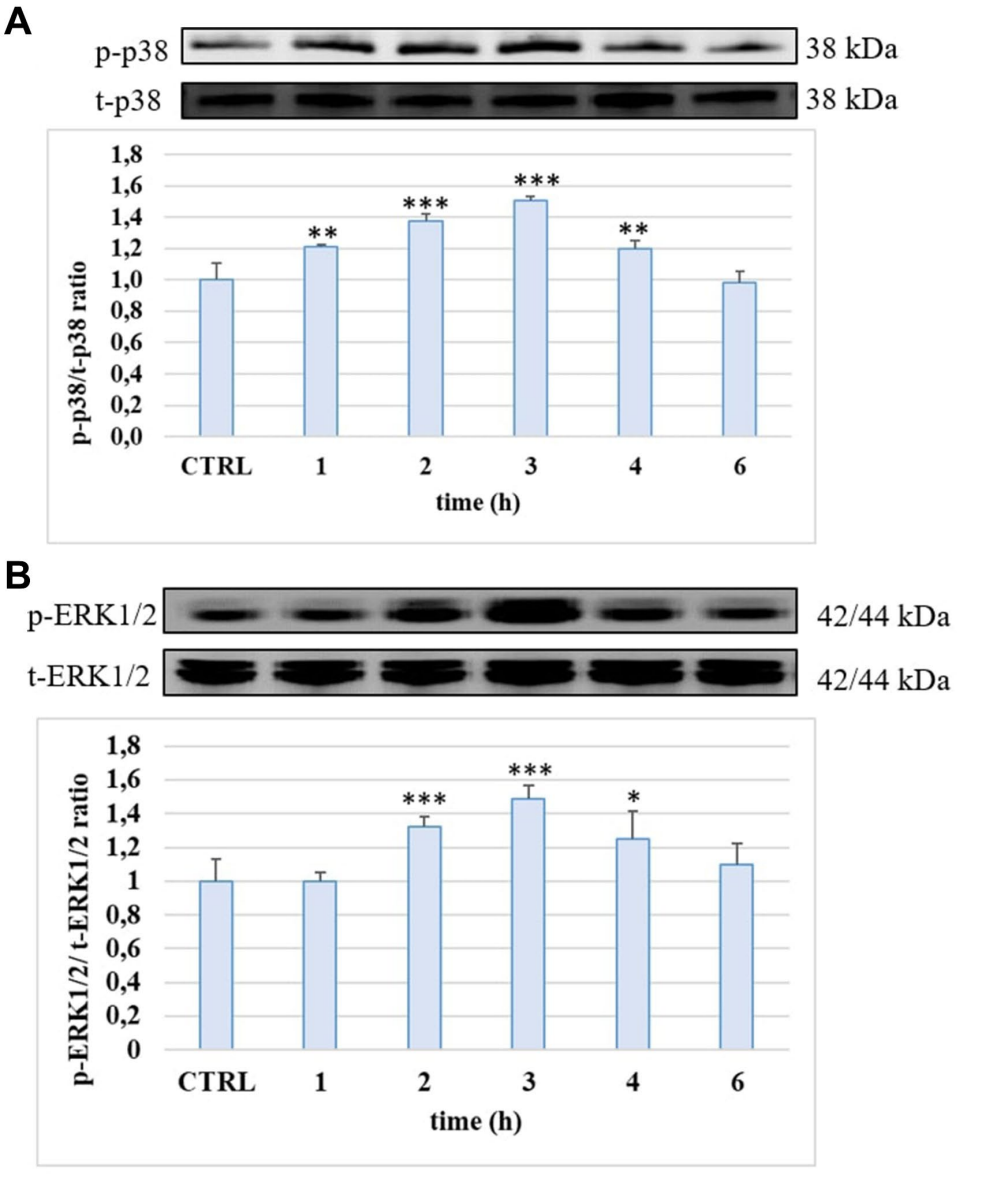
p-p38/t-p38 **(A)** and p-ERK1/2/t-ERK1/2 **(B)** ratio measured in HUVEC cells not treated (CTRL) or treated with LPS 10 μg/mL at different incubation time. ** = *p* < 0.01; *** = *p* < 0.001 LPS vs. CTRL (*n* = 3). Representative WB images of the experiment are shown.

The ability of the tested compounds to modulate some LPS-activated proinflammatory signaling pathways was evaluated by Western blot as analyses of the phosphorylation state of p38 and ERK1/2 MAPKs. The cells were treated with LPS (10 μg/mL) for 1, 2, 3, 4 and 6 h. Phosphorylation levels of p38 and ERK 1/2 detected in HUVEC cells treated with LPS is reported in Figure 5. LPS induced a significant phosphorylation of both proteins compared to the CTRL and at 3 h of incubation the activation reached the statistically significant highest values (about 50% for p-p38 and about 40% for p-ERK1/2, *p* < 0.001). At 6 h, the level of both MAPKs was led back to the CTRL values.

**Figure 6:**
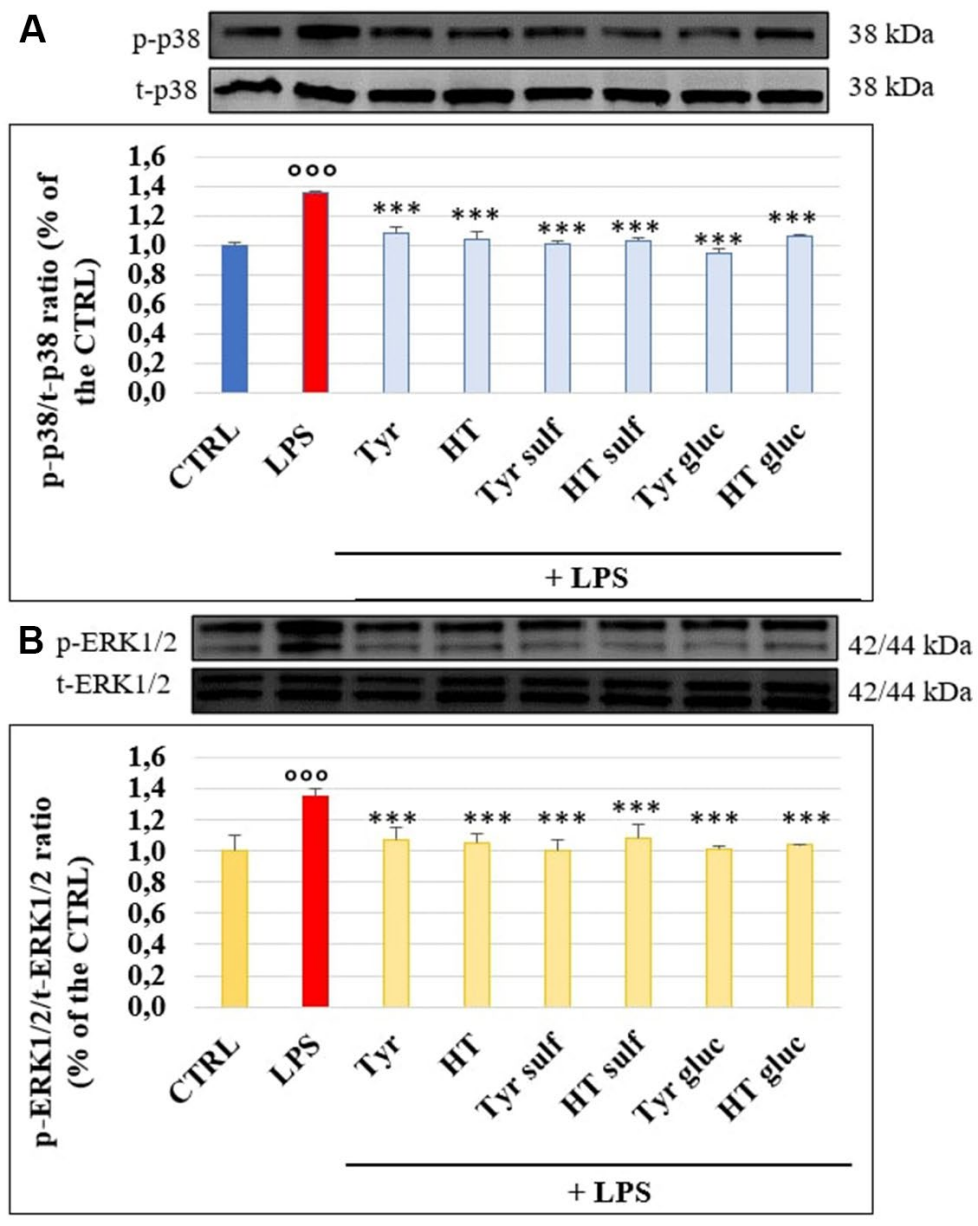
p-p38/t-p38 **(A)** and p-ERK1/2/t-ERK1/2 **(B)** measured in HUVEC cells pretreated with Tyr, HT, Tyr sulf, HT sulf, Tyr gluc, HT gluc (1 μM) or with an equivalent volume of MeOH (CTRL, 0 μM), and treated with LPS (10 μg/mL) for 3 h. °°° = *p* < 0.001 LPS vs. CTRL; *** = *p* < 0.001 Compounds vs. LPS (*n* = 3). Representative WB images of the experiment are shown.

Figures 6A,B shows p38 and ERK1/2 phosphorylation level and the effects exerted by phenolic compounds and their metabolites on HUVEC cells. As expected, LPS was able to significantly enhance the levels of p-p38 and p-ERK1/2 compared to untreated samples (CTRL) after 3 h of incubation, when the p-p38/t-p38 and p-ERK1/2/t-ERK1/2 ratios were about 35–40% higher in LPS-treated samples, compared to CTRL (100%). Pretreatment with compounds and metabolites significantly limited proteins phosphorylation and the efficacy were similar for all tested phenolic compounds (*p* > 0.05 compound vs. compound).

### 3.5 Modulation of NLRP3 inflammasome

**Figure 7:**
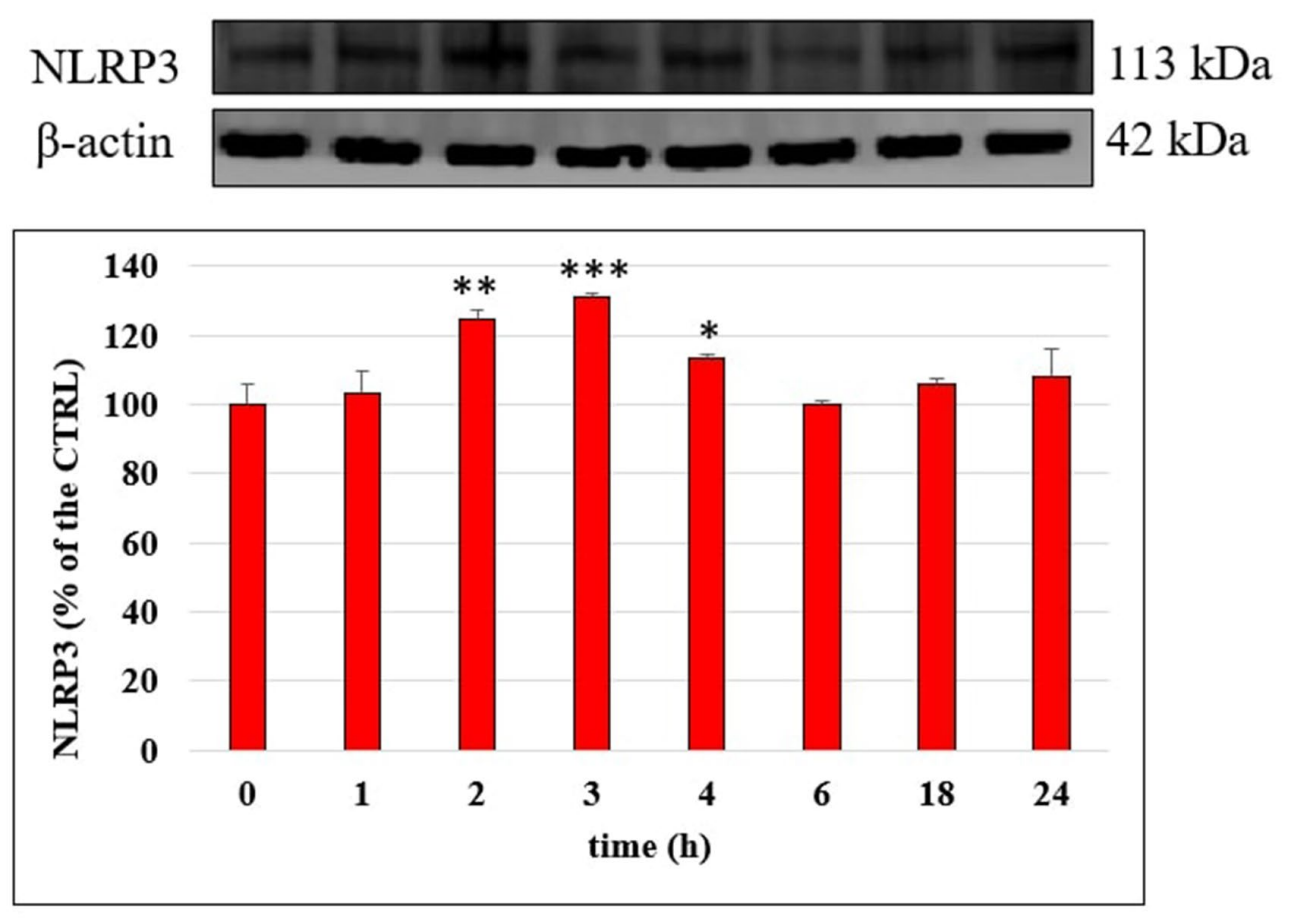
NLRP3/β-actin ratio measured in HUVEC cells not treated (CTRL) or treated with LPS 10 μg/mL at different incubation time. Data are reported as percentage compared to CTRL for each time. * = *p* < 0.05; ** = *p* < 0.01; *** = *p* < 0.001 LPS vs. CTRL (*n* = 3). Representative WB images of the experiment are shown.

**Figure 8:**
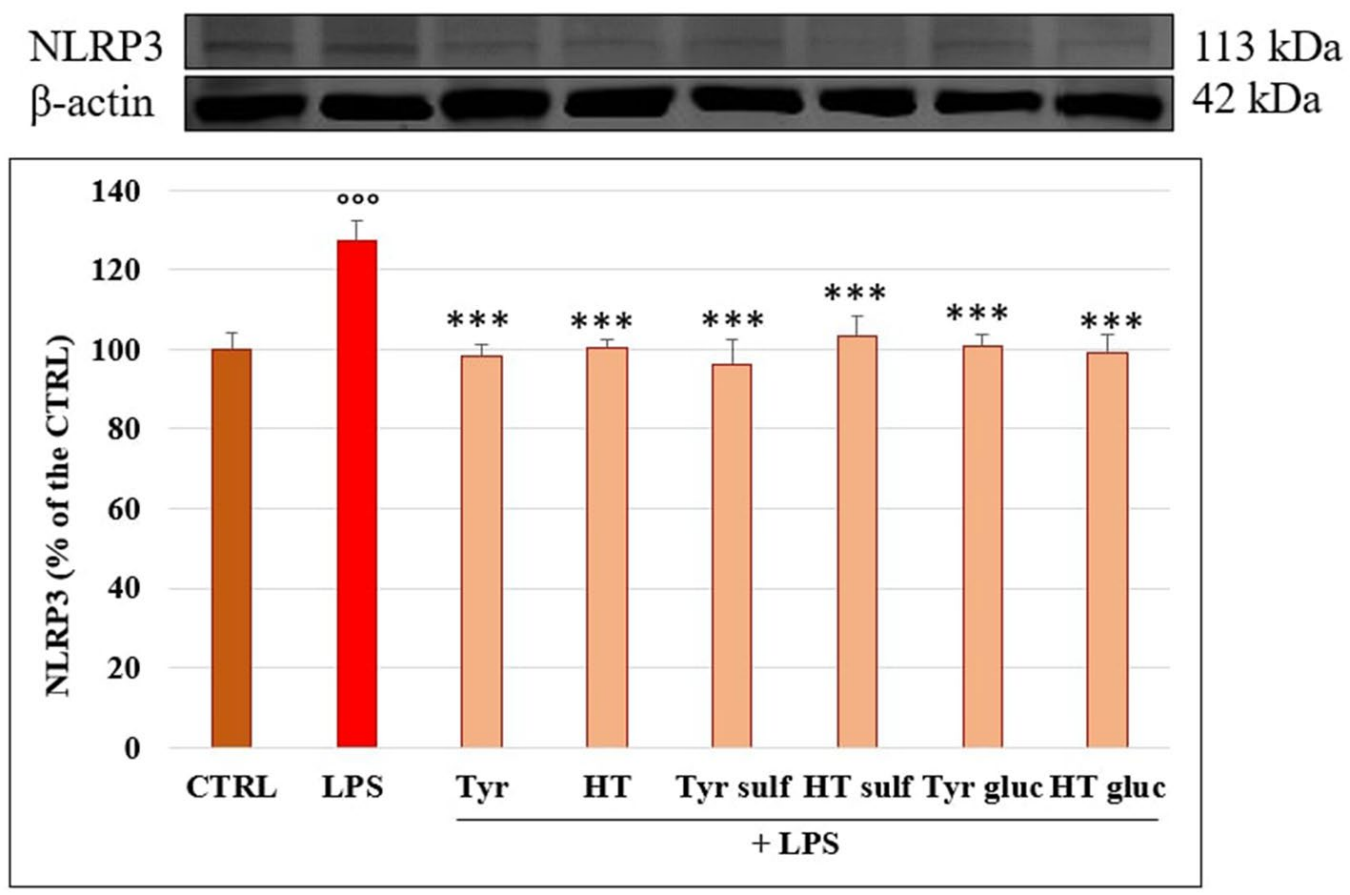
NLRP3/β-actin ratio measured in HUVEC cells pretreated with Tyr, HT, Tyr sulf, HT sulf, Tyr gluc, HT gluc (1 μM) or with an equivalent volume of MeOH (CTRL, 0 μM), and treated with LPS (10 μg/mL) for 3 h. °°° = *p* < 0.001 LPS vs. CTRL; *** = *p* < 0.001 Compounds vs. LPS (*n* = 3). Representative WB images of the experiment are shown.

NLRP3 inflammasome plays a fundamental role in the inflammatory response and in the mechanism of membrane permeability alteration at endothelial level; we therefore investigated its modulation by LPS on HUVEC monolayers with time through Western blotting. Figure 7 reports NLRP3 level in cells treated with LPS (10 μg/mL), for 1, 2, 3, 4, 6, 18 and 24 h; a significant increase of NLRP3 protein was observed mostly after 2 h (*p* < 0.01) and 3 h (*p* < 0.001) of incubation compared to CTRL. Starting from 6 h of incubation the NLRP3 protein level was led back to the CTRL value. The ability of Tyr, HT and their metabolites (1 μM) to modulate LPS induced increase of NLRP3 protein level was then evaluated in HUVEC cells after 3 h of incubation together with LPS (Figure 8). LPS treatment induced a significant increase (about 30%) of NLRP3 expression and pretreatment with all phenolic compounds significantly inhibited this rise. The observed protective effect was almost equal for all tested phenolic compounds, with no significant differences (*p* > 0.05).

## 4 Discussion

In this study we demonstrated for the first time the ability of the main HT and Tyr phase-II metabolites to limit the proinflammatory and proatherogenic effects induced by LPS (10 μg/mL) at endothelial level in an *in vitro* model of HUVEC cells. These sulphated and glucuronidated metabolites, usually originated by intestinal and hepatic biotransformation of dietary derived HT and Tyr, have been shown to reach systemic concentrations in the micromolar range, compatible with a biological activity (15, 19–21) and circulate at higher concentrations than their precursor compounds. Thus, it is likely that these conjugated forms perform *in vivo* the protective action usually attributed to their unconjugated precursors taken with the diet, and it is therefore meaningful to take into account their biological effects. Previous studies *in vitro* and *ex-vivo* already highlighted the ability of conjugated metabolites of HT and Tyr to exert protective actions against endothelial dysfunction (22–25).

Herein, to assess alteration of HUVEC monolayers permeability following LPS treatment we measured the leakage of FITC-Dextran through the monolayer, in accordance to Wang et al. (26). Consistent with previous reports, we confirmed the role of LPS in slacking the linkage between endothelial cells (6), highlighting a temporary alteration of the monolayer permeability. The transient increase of permeability induced by LPS represents an acute phenomenon that can result in serious endothelial dysfunction (e.g., sepsis condition) (27, 28). LPS has also been reported to cause an alteration of endothelial barrier function in HUVEC through the formation of paracellular gaps and changes of the cytoskeleton structure (6), and the modification of the TJ proteins ZO-1 and occludin (7, 17). In our experimental conditions, LPS-induced derangement of HUVEC monolayers was due to a decrease in the level of TJ proteins primarily involved in the regulation of paracellular permeability of the endothelium, ZO-1, occludin and JAM-A (5), as shown by Western blot analyses. Loss of TJ proteins as the result of an acute or chronic process leads to a broad range of pathological conditions in humans including systemic capillary leak syndrome, angioedema, and other diseases characterized by altered paracellular permeability (4, 29). Here we show that the loss of the monolayer integrity was prevented by all the tested phenolic metabolites, that significantly reduced LPS-induced FITC-Dextran permeation. The protective action of the metabolites was comparable to that of the parent compounds and was due, at least in part, to their ability to inhibit TJ protein loss. HT and Tyr sulfated and glucuronidated metabolites have been in fact shown to modulate TJ proteins preserving their concentration with values similar to those shown by untreated cells. There are no other studies in the literature showing similar activity by single phenolic compounds or their metabolites in this experimental model. Additionally, there are very few studies that evaluated the effect of metabolites of phenolic compounds, and one of these highlighted a protective effect toward TJ proteins occluding and ZO-1 exerted by some of the metabolites of pterostilbene (30).

LPS-induced endothelial permeability and TJ proteins alteration has been reported to be a consequence of its binding to TLR4 on endothelial cell surface. This binding can activate several intracellular signaling pathways, such as the MAPKs and NF-𝜅B, that participate in endothelial inflammation by modulating the expression of adhesion molecules and pro-inflammatory cytokines (31, 32). Overall, several studies have well described how p38 and ERK1/2 MAPKs regulate the endothelial barrier function by modulating the expression of TJs proteins (33). Herein, our data confirm the involvement of the MAPK pathway in LPS-induced endothelial TJ alteration in HUVEC cultures, where an increase of phosphorylation state of p38 and ERK1/2 was observed in LPS treated samples with respect to the controls, as observed also in other cell lines (34). These findings suggest that the tested phenolic compounds exhibit their protective effect on LPS-induced injury, at least in part, through their ability to modulate MAPK signaling pathways, in agreement with other studies regarding EVOO phenolics and their capacity of down-regulating MAPK activity in endothelial cells (24). Another study, although in a different cell type, has recently shown how the metabolites of HT and Tyr are active in counteracting the effect of LPS on the activation of MAPKs, thus inhibiting the expression of a proinflammatory phenotype (35, 36).

Among cellular responses modulated by MAPK signaling, the activation of NLRP3 inflammasome (37) has been the focus of recent research, since its role in the onset and progression of atherosclerosis has been highlighted (38). In our experimental conditions, LPS treatment triggered in HUVEC a significant increase of the level of the NLRP3 complex and, for the first time, we highlighted the ability of sulphated and glucuronidated metabolites of Tyr and HT to modulate its activation. The inhibition of NLRP3 activation has been postulated as one of the possible mechanisms of the anti-inflammatory action of dietary phenolic compounds, as reviewed by Blevins et al. (39).

Taken together our data strengthen the hypothesis that circulating HT and Tyr metabolites may exert a significant role in the maintenance of endothelial barrier integrity. The efficacy of these compounds was evaluated at physiologically relevant concentrations and at the moment of greatest evidence of the proinflammatory activity of LPS based on the incubation curves. These experimental conditions were chosen to obtain data that can be more easily translated to what happens *in vivo* and represent a strength of this investigation. Nonetheless, one of the limitations is related to the cellular model used in this study, which does not present all the cell types usually found in the blood stream [e.g., red blood cells (RBC)], all able to affect compounds’ bioavailability *in vivo* (40). Moreover, it is not known yet if metabolites are able to exert a biological action themselves or if they work just as “pro-drugs” of their free forms, representing another limitation of this study. It has been reported that metabolites may undergo deconjugation before entering the cells, releasing free forms which may be partially converted into other metabolites inside the cell environment, acting as conjugated forms (40). Thus, a continually changing pool of parental free forms and their major *in vivo* formed metabolites, may be responsible, as a whole, for the observed beneficial effect of EVOO phenolic fraction in the prevention and amelioration of endothelial damage leading to cardiovascular diseases. For this purpose, a further investigation will be needed to evaluate the metabolites coming from endothelial and other circulating cells metabolism, which may effectively act on the molecular mechanisms analyzed in this study. However, our preliminary findings enlarge the existing evidence regarding the role of phenolic compounds metabolites and contribute to understand the *in vivo* cardioprotective effects of EVOO dietary interventions.

## Data availability statement

The original contributions presented in the study are included in the article/Supplementary material, further inquiries can be directed to the corresponding author.

## Ethics statement

Ethical approval was not required for the studies on humans in accordance with the local legislation and institutional requirements because only commercially available established cell lines were used. Ethical approval was not required for the studies on animals in accordance with the local legislation and institutional requirements because only commercially available established cell lines were used.

## Author contributions

SZ: Data curation, Formal analysis, Investigation, Methodology, Writing – original draft. GS: Data curation, Formal analysis, Supervision, Validation, Writing – original draft, Writing – review & editing. MM: Supervision, Visualization, Writing – review & editing. BF: Formal analysis, Visualization, Writing – original draft. AB: Data curation, Formal analysis, Writing – review & editing. RT: Conceptualization, Funding acquisition, Supervision, Writing – review & editing. MD: Conceptualization, Data curation, Funding acquisition, Project administration, Supervision, Visualization, Writing – original draft.

## Supplementary material

SUPPLEMENTARY FIGURE S1 Structures of HT, Tyr and their main phase II metabolites tested in this study.

SUPPLEMENTARY FIGURE S2 Cell viability, expressed as % of control values (100%), measured with the MTT assay, in HUVEC cells exposed to EVOO phenolic compounds and their metabolites (0.5, 1, and 2.5 μM) for 24 h or with an equivalent volume of MeOH (CTRL, 0 μM). HT = hydroxytyrosol; Tyr = tyrosol; HT gluc = hydroxytyrosol-3-glucuronide, HT sulf = hydroxytyrosol-3-sulfate, Tyr gluc = tyrosol-glucuronide; Tyr sulf = tyrosol-sulfate. Data are presented as average ± SD. *p* > 0.05 vs. control (*n* = 12).

SUPPLEMENTARY FIGURE S3 Cell viability, expressed as % of control values (100%), measured with the MTT assay, in HUVEC cells pre-treated with EVOO phenolic compounds and their metabolites (0.5, 1 and 2.5 μM) or with an equivalent volume of MeOH (CTRL, 0 μM), and co-treated with LPS (10 μg/mL) for 24 h. Data are presented as average ± SD. *p* > 0.05 vs. control (*n* = 12).
