# Supplementary figures and images for: Protective effect of hydroxytyrosol and tyrosol metabolites in LPS-induced vascular barrier derangement in vitro
Source: Front Nutr. 2024 Apr 19;11:1350378. doi: 10.3389/fnut.2024.1350378 (PMC11066181; doi:10.3389/fnut.2024.1350378)

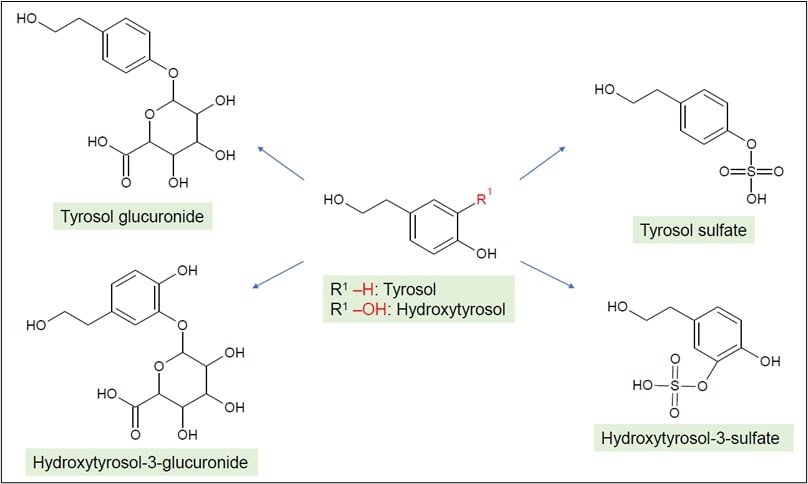

Supplement: SUPPLEMENTARY FIGURE S1 — Structures of HT, Tyr and their main phase II metabolites tested in this study. [file Image_1.JPEG]

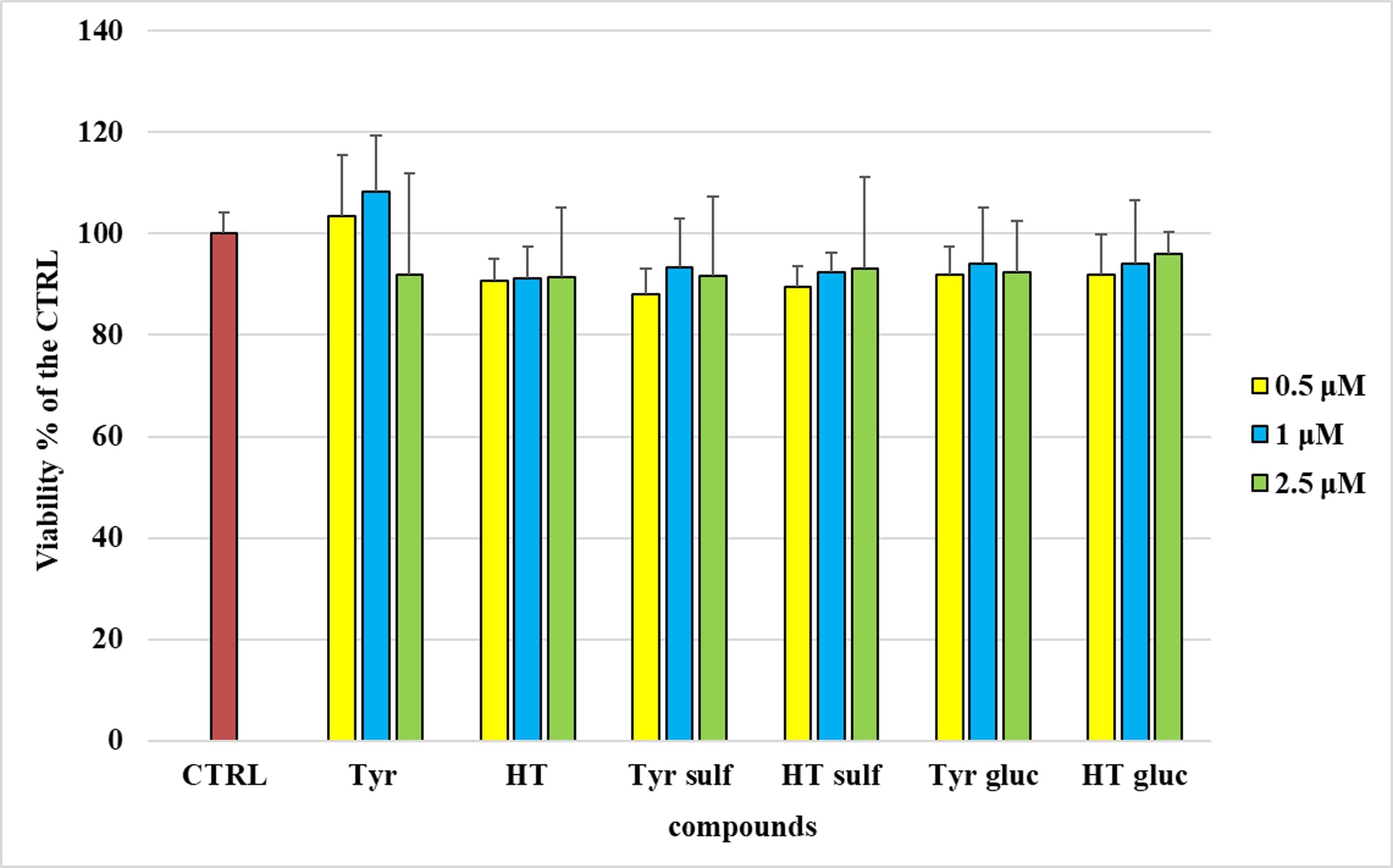

Supplement: SUPPLEMENTARY FIGURE S2 — Cell viability, expressed as % of control values (100%), measured with the MTT assay, in HUVEC cells exposed to EVOO phenolic compounds and their metabolites (0.5, 1, and 2.5 μM) for 24 h or with an equivalent volume of MeOH (CTRL, 0 μM). HT = hydroxytyrosol; Tyr = tyrosol; HT gluc = hydroxytyrosol-3-glucuronide, HT sulf = hydroxytyrosol-3-sulfate, Tyr gluc = tyrosol-glucuronide; Tyr sulf = tyrosol-sulfate. Data are presented as average ± SD. p > 0.05 vs. control (n = 12). [file Image_2.JPEG]

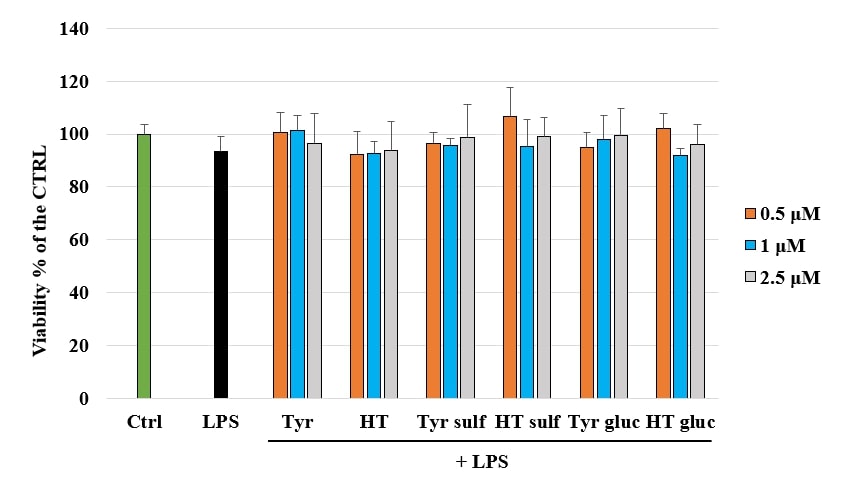

Supplement: SUPPLEMENTARY FIGURE S3 — Cell viability, expressed as % of control values (100%), measured with the MTT assay, in HUVEC cells pre-treated with EVOO phenolic compounds and their metabolites (0.5, 1 and 2.5 μM) or with an equivalent volume of MeOH (CTRL, 0 μM), and co-treated with LPS (10 μg/mL) for 24 h. Data are presented as average ± SD. p > 0.05 vs. control (n = 12). [file Image_3.JPEG]
